# Supplementary material for: Geospatial assessment of household water, sanitation and hygiene conditions and associated factors in Nigeria: A causal relationship model
Source: PLoS One. 2025 Aug 14;20(8):e0330167. doi: 10.1371/journal.pone.0330167 (PMC12352836; doi:10.1371/journal.pone.0330167)
Supplement: S1 File — (DOCX) [file pone.0330167.s001.docx]

Table 1: Sociodemographic characteristics of older adults in Nigeria

| Characteristics of the respondents | Frequency (N=40,369) | Percentage |
| --- | --- | --- |
| **Age of household head** |  |  |
| <60 | 31,992 | 79.3 |
| 60+ | 8377 | 20.7 |
| **Sex of the household head** |  |  |
| Male | 33088 | 82.0 |
| Female | 7281 | 18.0 |
| **Household wealth status** |  |  |
| Poorest | 6903 | 17.1 |
| Poorer | 7485 | 18.5 |
| Middle | 8262 | 20.5 |
| Richer | 8666 | 21.5 |
| Richest | 9053 | 22.4 |
| **Household level of education** |  |  |
| Low | 13585 | 33.7 |
| Middle | 13432 | 33.3 |
| High | 13352 | 33.0 |
| **Household size** |  |  |
| 6 or less | 31505 | 78.0 |
| More than 6 members | 8864 | 22.0 |
| **Type of place of residence** |  |  |
| Urban | 18899 | 46.8 |
| Rural | 21470 | 53.2 |
| **Region** |  |  |
| North central | 5695 | 14.1 |
| North East | 5694 | 14.1 |
| North West | 9816 | 24.3 |
| South East | 4746 | 11.8 |
| South South | 5715 | 14.2 |
| South West | 8703 | 21.6 |

^a^ there are some missing responses

**Table 2. Household characteristics of older adults by state**

| **State** | **Household head aged 60+ years** | **Female-headed households** | **Households below middle wealth quintile** | **Households with low level of education** | **Households with 6+ members** | **Households with rural residence** | **Totals** |
| --- | --- | --- | --- | --- | --- | --- | --- |
| Abia | 29.5 | 22.9 | 2.0 | 11.1 | 15.2 | 80.0 | 663 |
| Adamawa | 20.6 | 12.7 | 54.2 | 47.4 | 21.8 | 76.2 | 908 |
| Anambra | 26.4 | 26.3 | 7.7 | 12.0 | 16.8 | 14.2 | 1391 |
| Akwa-Ibom | 19.7 | 28.6 | 17.8 | 13.1 | 13.0 | 92.0 | 1048 |
| Bauchi | 18.1 | 3.8 | 72.4 | 63.6 | 41.7 | 86.3 | 1146 |
| Bayelsa | 19.4 | 30.5 | 14.9 | 9.1 | 14.3 | 67.2 | 404 |
| Benue | 15.6 | 22.0 | 45.6 | 22.4 | 16.1 | 81.5 | 1230 |
| Borno | 21.8 | 15.1 | 41.3 | 55.6 | 30.9 | 47.4 | 1271 |
| Cross-River | 19.7 | 32.1 | 30.6 | 15.0 | 7.4 | 80.7 | 739 |
| Delta | 25.2 | 27.1 | 5.8 | 9.0 | 10.8 | 43.9 | 1259 |
| Ebonyi | 21.5 | 31.5 | 47.2 | 32.0 | 22.9 | 15.2 | 851 |
| Edo | 23.1 | 29.3 | 15.6 | 15.2 | 13.8 | 39.1 | 709 |
| Ekiti | 31.6 | 29.3 | 23.8 | 11.6 | 8.4 | 18.3 | 628 |
| Enugu | 32.8 | 34.4 | 19.9 | 15.7 | 13.1 | 22.8 | 870 |
| Gombe | 17.1 | 4.6 | 69.9 | 61.0 | 43.6 | 75.6 | 550 |
| Imo | 31.7 | 32.7 | 2.7 | 11.6 | 21.2 | 38.1 | 971 |
| Jigawa | 17.7 | 2.8 | 84.5 | 68.5 | 40.0 | 88.2 | 1134 |
| Kaduna | 14.7 | 7.9 | 34.9 | 41.7 | 31.4 | 57.8 | 1949 |
| Kano | 21.2 | 14.1 | 56.6 | 51.6 | 37.6 | 56.3 | 2153 |
| Katsina | 22.3 | 7.4 | 64.2 | 55.0 | 39.6 | 78.4 | 1820 |
| Kebbi | 14.7 | 9.4 | 74.8 | 81.0 | 35.8 | 83.7 | 910 |
| Kogi | 24.6 | 37.0 | 24.9 | 24.5 | 12.5 | 60.3 | 707 |
| Kwara | 28.3 | 26.5 | 29.3 | 39.1 | 16.6 | 24.4 | 770 |
| Lagos | 14.4 | 15.7 | 0.4 | 6.2 | 9.0 | 2.9 | 2965 |
| Nasarawa | 18.3 | 11.8 | 21.6 | 23.3 | 24.3 | 65.7 | 590 |
| Niger | 15.0 | 2.6 | 47.2 | 56.0 | 29.8 | 74.5 | 1229 |
| Ondo | 26.1 | 25.9 | 23.2 | 13.5 | 10.3 | 46.3 | 843 |
| Ogun | 18.6 | 25.1 | 7.6 | 19.3 | 5.3 | 49.4 | 1251 |
| Osun | 26.1 | 24.7 | 23.6 | 17.4 | 5.7 | 23.9 | 1219 |
| Oyo | 26.3 | 24.7 | 14.8 | 22.9 | 6.8 | 22.4 | 1797 |
| Plateau | 19.9 | 18.3 | 48.3 | 21.2 | 20.7 | 67.3 | 883 |
| Rivers | 10.9 | 25.8 | 7.8 | 5.0 | 9.2 | 48.2 | 1556 |
| Sokoto | 21.8 | 3.6 | 80.1 | 85.0 | 32.7 | 82.6 | 819 |
| Taraba | 17.8 | 11.8 | 66.0 | 40.0 | 32.8 | 79.0 | 720 |
| Yobe | 22.9 | 8.0 | 81.9 | 76.2 | 40.6 | 79.8 | 1100 |
| Zamfara | 19.3 | 1.2 | 75.0 | 70.4 | 36.7 | 75.0 | 1030 |
| FCT-Abuja | 10.1 | 16.4 | 15.0 | 18.5 | 20.8 | 28.5 | 287 |
| **Nigeria (Overall data)** | **20.8** | **18.0** | **35.6** | **33.7** | **22.0** | **53.2** | **40,369** |

**Overview of unimproved WASH in Nigeria**

The analysis of the unimproved WASH indicated that more than three-quarter (83%) of the households were using at least one of the unimproved water, sanitation and hygiene during the study period (Figure 1). About 16% of the households were using all the three unimproved WASH, and about 30% reported unimproved water and hygiene or water and sanitation or hygiene and sanitation. Overall, unimproved hygiene was the most reported (77%), nearly half (44%) reported unimproved sanitation and just one-quarter reported using unprotected water (Table 1). However, there are variations across the states and regions.


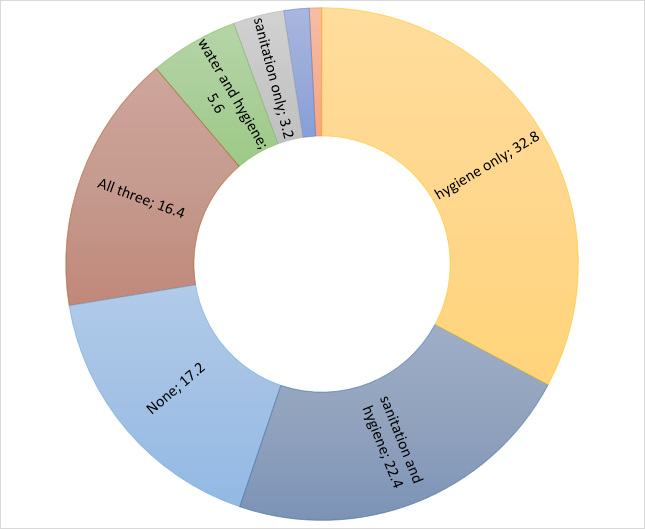


Water & sanitation; 1.6

Water only; 0.8

Figure 1: Prevalence of unimproved water, sanitation and hygiene among households in Nigeria

**Table 3. Variations in WASH condition of households by states in Nigeria**

| State | Unimproved water | Unimproved sanitation | Unimproved hygiene | Totals |
| --- | --- | --- | --- | --- |
| Abia | 73 (11.0) | 50 (7.6) | 262 (39.6) | 663 |
| Adamawa | 432 (47.5) | 222 (24.4) | 894 (98.4) | 908 |
| Anambra | 131 (9.4) | 249 (17.9) | 598 (43.0) | 1391 |
| Akwa-Ibom | 195 (18.6) | 128 (12.2) | 969 (92.4) | 1048 |
| Bauchi | 438 (38.2) | 784 (68.4) | 1111 (96.9) | 1146 |
| Bayelsa | 127 (31.3) | 285 (70.4) | 357 (88.2) | 404 |
| Benue | 259 (21.1) | 657 (53.4) | 1214 (98.7) | 1230 |
| Borno | 358 (28.1) | 463 (36.4) | 1207 (94.9) | 1271 |
| Cross-River | 316 (42.7) | 377 (51.0) | 393 (53.2) | 739 |
| Delta | 222 (17.7) | 423 (33.6) | 877 (69.7) | 1259 |
| Ebonyi | 191 (22.5) | 701 (82.4) | 826 (97.1) | 851 |
| Edo | 74 (10.4) | 205 (29.0) | 622 (87.7) | 709 |
| Ekiti | 66 (10.5) | 287 (45.6) | 545 (86.8) | 628 |
| Enugu | 176 (20.2) | 520 (59.8) | 755 (86.8) | 870 |
| Gombe | 305 (55.6) | 144 (26.3) | 435 (79.2) | 550 |
| Imo | 45 (4.6) | 102 (10.5) | 797 (82.1) | 971 |
| Jigawa | 182 (16.1) | 967 (85.3) | 1111 (98.0) | 1134 |
| Kaduna | 559 (28.7) | 621 (31.9) | 1844 (94.6) | 1949 |
| Kano | 752 (34.9) | 874 (40.6) | 1761 (81.8) | 2153 |
| Katsina | 670 (36.8) | 1188 (65.3) | 725 (39.9) | 1820 |
| Kebbi | 461 (50.7) | 713 (78.3) | 903 (99.2) | 910 |
| Kogi | (196 (27.7) | 506 (71.6) | 553 (78.2) | 707 |
| Kwara | 215 (27.9) | 431 (55.9) | 744 (96.6) | 770 |
| Lagos | 17 (0.6) | 435 (14.7) | 1669 (56.3) | 2965 |
| Nasarawa | 115 (19.4) | 147 (24.8) | 483 (81.9) | 590 |
| Niger | 452 (36.8) | 727 (59.2) | 1179 (96.0) | 1229 |
| Ondo | 178 (21.1) | 419 (49.7) | 824 (97.7) | 843 |
| Ogun | 152 (12.2) | 273 (21.9) | 946 (75.6) | 1251 |
| Osun | 170 (13.9) | 474 (38.9) | 302 (24.8) | 1219 |
| Oyo | 200 (11.1) | 689 (38.3) | 880 (49.0) | 1797 |
| Plateau | 400 (45.4) | 521 (59.0) | 703 (79.6) | 883 |
| Rivers | 89 (5.7) | 606 (38.9) | 960 (61.7) | 1556 |
| Sokoto | 537 (65.6) | 461 (56.2) | 809 (98.8) | 819 |
| Taraba | 349 (48.5) | 337 (46.8) | 702 (97.6) | 720 |
| Yobe | 303 (27.6) | 787 (71.6) | 10113 (92.1) | 1100 |
| Zamfara | 443 (43.1) | 775 (75.2) | 990 (96.1) | 1030 |
| FCT-Abuja | 34 (11.8) | 73 (25.5) | 196 (68.5) | 287 |
| **Nigeria (Overall data)** | **9881 (24.5)** | **17619 (43.7)** | **31162 (77.2)** | **40,369** |

*Bivariate analysis*

The result in Table 4 indicated that at the time of the survey for this study, 25% of Nigerian households utilise unprotected water for drinking, 47% were using unimproved sanitation while 88% were using unimproved hygiene in the household. According to the result, a higher proportion of households headed by male used unprotected water (26%) compared to female headed households (18%). Utilization of unprotected water was also more prominent among rural households (37%) compared to urban residents (8%). Similarly, utilization of unprotected drinking water was highest among the households in the poorest wealth quintile (54%), followed by the poorer households (40%); households with low level of education (41%) and those with larger household size of more than six members (31%). The least proportion was observed among the households in the richest wealth quintile (1%), high level of education (11%) and small (less than six) family size (13%). Proportion of households utilising unsafe drinking water was higher in the Northern regions (29 – 35%) compared to the households in Southern Nigeria (11 – 18%).

The result further indicated that use of unimproved sanitation was also associated with all the selected household characteristics except the age of the household head (p<0.05). Utilization of unimproved sanitation was more prevalent among households in rural areas (62%) compared to urban (28%) and male headed households compared to the female headed ones (50% versus 44%); those in the lower (poorest/poorer/middle) wealth quintiles (48 – 89%) compared to those in the upper (richer/richest) quintiles (4 – 23%); households with low level of education (67%) compared to those with high level of education (27%); larger households (51%) compared to the smaller ones (46%); Northern households (49 – 59%) compared to their Southern counterparts (35 – 39%).

Utilization of unimproved hygiene was associated with only the households’ rural-urban residence, wealth quintile, level of education and region of residence (p<0.05). Though the proportion of households with unimproved hygiene was generally high irrespective of household peculiarity, it was much highest among rural households (91%) compared to urban (83%), poorest households (95%) compared to the richest (68%), low-education households (93%) compared the high-education ones (80%) and in Northern Nigeria (86 – 97%) compared to the households in the South (75 – 89%).

Table 4. Chi-Square test showing bivariate association between household factors and unimproved water, sanitation and hygiene (WASH) among older adults in Nigeria

| **Household characteristics** | Unprotected Water | | Unimproved sanitation | | | Unimproved hygiene | | |  |
| --- | --- | --- | --- | --- | --- | --- | --- | --- | --- |
|  | n(%) | χ^2^ | n(%) | χ^2^ | n(%) | | χ^2^ | Total ^c^ | |
| **Sex of household head** |  |  |  |  |  | |  |  | |
| Male | 8609 (26.0) | 69.91*** | 14677 (44.4) | 13.40*** | 25584 (77.3) | | 0.77 | 33088 (82.0) | |
| Female | 1272 (17.5) |  | 2942 (40.4) |  | 5578 (76.6) | |  | 7281 (18.0) | |
| **Age of household head** |  |  |  |  |  | |  |  | |
| <60 | 7729 (24.2) | 4.44* | 13760 (43.0) | 11.26*** | 24619 (77.0) | | 2.11 | 31992 (79.3) | |
| 60+ | 2152 (25.7) |  | 3859 (46.1) |  | 6543 (78.1) | |  | 8377 (20.7) | |
| **Household wealth status** |  |  |  |  |  | |  |  | |
| Poorest | 4001 (58.0) | 479.25*** | 6,113 (88.6) | 436.60*** | 6487 (94.0) | | 304.16*** | 6903 (17.1) | |
| Poorer | 3273 (43.7) |  | 5,186 (69.3) |  | 6703 (89.6) | |  | 7485 (18.5) | |
| Middle | 1938 (23.5) |  | 3918 (47.4) |  | 7049 (85.3) | |  | 8262 (20.5) | |
| Richer | 583 (6.7) |  | 2063 (23.8) |  | 6529 (75.3) | |  | 8666 (21.5) | |
| Richest | 86 (0.9) |  | 340 (3.8) |  | 4394 (48.5) | |  | 9053 (22.4) | |
| **Household educational level** ^a^ |  |  |  |  |  | |  |  | |
| Low | 5814 (42.8) | 368.86*** | 8914 (65.6) | 404.49*** | 12258 (90.2) | | 310.16*** | 13583 (33.7) | |
| Middle | 2687 (20.0) |  | 5586 (41.6) |  | 10362 (77.1) | |  | 13433 (33.3) | |
| High | 1380 (10.3) |  | 3119 (23.4) |  | 8542 (64.0) | |  | 13353 (33.1) | |
| **Household size** |  |  |  |  |  | |  |  | |
| <6 | 7015 (22.3) | 120.27*** | 13220 (42.0) | 41.24*** | 23878 (75.8) | | 65.39*** | 31505 (78.0) | |
| >6 | 2866 (32.3) |  | 4399 (49.6) |  | 7284 (82.2) | |  | 8864 (22.0) | |
| **Household’s residence type** |  |  |  |  |  | |  |  | |
| Urban | 1466 (7.8) | 302.98*** | 4646 (24.6) | 237.44*** | 12754 (67.5) | | 285.57*** | 18899 (46.8) | |
| Rural | 8415 (39.2) |  | 12973 (60.4) |  | 18408 (85.7) | |  | 21470 (53.2) | |
| **Region** |  |  |  |  |  | |  |  | |
| North central | 1671 (29.3) | 37.35*** | 3062 (53.8) | 24.87*** | 5073 (89.1) | | 102.61*** | 5695 (14.1) | |
| North East | 2184 (38.4) |  | 2736 (48.1) |  | 5362 (94.2) | |  | 5694 (14.1) | |
| North West | 3606 (36.7) |  | 5599 (57.0) |  | 8144 (83.0) | |  | 9816 (24.3) | |
| South East | 616 (13.0) |  | 1623 (34.2) |  | 3239 (68.2) | |  | 4746 (11.8) | |
| South South | 1022 (17.9) |  | 2024 (35.4) |  | 4177 (73.1) | |  | 5716 (14.2) | |
| South West | 783 (9.0) |  | 2576 (29.6) |  | 5166 (59.4) | |  | 8703 (21.6) | |
| **Total** | **9881 (24.5)** | | **17619 (43.6)** | | **31162 (77.2)** | | | **40369** | |

*** p<0.001; ** p<0.01; * p<0.05

^c^ Column percentage of the total

**Factors associated with unimproved WASH in Nigeria**

*Multivariate analysis*

The results in Table 5 showed the multivariate analysis of the household characteristics associated with use of unimproved water, sanitation and hygiene in Nigerian households. According to the results, household headed by female had 27% significantly lower odds of using unprotected water (OR=0.73, p<0.001, 95% C.I.=0.64 – 0.83) and 17% lower odds of using unimproved sanitation (OR=0.83, p<0.001, 95% C.I.=0.76 – 0.91), relative to households with male heads. In households headed by older adults aged 60 years and above, the odds of using unimproved sanitation was 8% significantly lower (OR=0.92, p<0.05, 95% C.I.=0.88–1.10) compared to households headed by persons below age 60 years.

Households with richer/richest wealth status had 86%, 90% and 59% significantly lower odds of using unprotected water, unimproved sanitation and unimproved hygiene respectively, compare to their counterparts from poorest/poorer/middle households. Similarly, relative to households with low level of education, the odds of utilizing unprotected water, unimproved sanitation and hygiene was 36%, 32% and 23% respectively lower among households with middle level of education. Households with high level of education showed a similar result, with much lower odds of unimproved WASH, compared to the households with low level of education. Larger households were more prone to unsafe water but less of unimproved hygiene conditions than the small-sized ones. For instance, the result showed that lager households with six or more members had 9% higher odds of utilizing unimproved water but 14% lower odds for unimproved hygiene, compared to smaller households with less than six members.

The result further revealed that rural households had about three-fold significantly higher odds of using unprotected water, and two-fold higher odds of using unimproved sanitation with reference to urban households. The odds of using unimproved sanitation was significantly lower only among households in the North East and North West by 66% and 44% respectively with reference to the households in the North Central. For unimproved hygiene, households in all the other regions except North East had 56 – 82% lower odds, relative to those in the North Central.

Table 5. Binary Logistic regression showing the household factors that predict the unimproved WASH condition in Nigerian households at multivariate level

| **Household characteristics** | Unprotected Water | Unimproved sanitation | Unimproved hygiene |
| --- | --- | --- | --- |
|  | Adjusted OR (95% C.I.) | Adjusted OR (95% C.I.) | Adjusted OR (95% C.I.) |
| **Sex of household head** |  |  |  |
| Male ^RC^ | 1.00 | 1.00 | 1.00 |
| Female | 0.72 (0.64 – 0.82)*** | 0.84 (0.77 – 0.92)*** | 1.11 (1.01 – 1.21)* |
| **Age of household head** |  |  |  |
| <60 ^RC^ | 1.00 | 1.00 | 1.00 |
| 60+ | 1.01 (0.93 – 1.09) | 0.91 (0.84 – 0.99)* | 0.95 (0.87 – 1.03) |
| **Household wealth status** |  |  |  |
| Poorest/poorer/middle ^RC^ | 1.00 | 1.00 | 1.00 |
| Richer/Richest | 0.12 (0.10 – 0.14)*** | 0.10 (0.08 – 0.12)*** | 0.35 (0.31 – 0 39)*** |
| **Household educational level** |  |  |  |
| Low ^RC^ | 1.00 | 1.00 | 1.00 |
| Middle | 0.68 (0.59 – 0.79)*** | 0.69 (0.62 – 0.78)*** | 0.69 (0.59 – 0.81)*** |
| High | 0.60 (0.51 – 0.71)*** | 0.49 (0.43 – 0.56)*** | 0.48 (0.41 – 0.57)*** |
| **Household Size** |  |  |  |
| <6 ^RC^ | 1.00 | 1.00 | 1.00 |
| >6 | 1.11 (1.01 – 1.21) * | 1.00 (0.92 – 1.09) | 0.95 (0.87 – 1.05) |
| **Type of place of residence** |  |  |  |
| Urban ^RC^ | 1.00 | 1.00 | 1.00 |
| Rural | 3.01 (2.30 – 3.96)*** | 2.06 (1.73 – 2.46)*** | 1.18 (1.04 – 1.34)* |
| **Region** |  |  |  |
| North central ^RC^ | 1.00 | 1.00 |  |
| North East | 0.92 (0.62 – 1.35) | 0.35 (0.27 – 0.45)*** | 1.35 (1.03 – 1.78)* |
| North West | 0.87 (0.63 – 1.20) | 0.57 (0.45 – 0.73)*** | 0.39 (0.32 – 0.49)*** |
| South East | 0.86 (0.57 – 1.28) | 0.89 (0.69 – 1.15) | 0.33 (0.27 – 0.41)*** |
| South South | 0.98 (0.69 – 1.41) | 0.87 (0.65 – 1.18) | 0.45 (0.37 – 0.56)*** |
| South West | 0.69 (0.46 – 1.04) | 1.09 (0.73 – 1.63) | 0.27 (0.22 – 0.34)*** |

RC Reference category; *** p<0.001; ** p<0.01; * p<0.05; OR odds ratio; C.I. confidence interval
