# Supplementary material for: Geospatial assessment of household water, sanitation and hygiene conditions and associated factors in Nigeria: A causal relationship model
Source: PLoS One. 2025 Aug 14;20(8):e0330167. doi: 10.1371/journal.pone.0330167 (PMC12352836; doi:10.1371/journal.pone.0330167)
Supplement: Attachment — Submitted filename: Authors Response.pdf [file pone.0330167.s004.pdf]

**GEOSPATIAL ASSESSMENT OF HOUSEHOLD WATER, SANITATION AND HYGIENE CONDITIONS AND ASSOCIATED FACTORS IN NIGERIA: A CAUSAL RELATIONSHIP MODEL**

Summarized authors' response: We have made all the corrections in accordance with the Editor's and Reviewers' suggestions. The paper has been formatted in line with the guidelines of the Journal. The modified/added sentences are colored red.

| S/No. | Academic Editor's Comments | Changes Made | Page Number |
|---|---|---|---|
| 1 | Please review your reference list to ensure that it is complete and correct. If you have cited papers that have been retracted, please include the rationale for doing so in the manuscript text, or remove these references and replace them with relevant current references. Any changes to the reference list should be mentioned in the rebuttal letter that accompanies your revised manuscript. If you need to cite a retracted article, indicate the article's retracted status in the References list and also include a citation and full reference for the retraction notice. | We have ensured that only cited references are listed. None of the cited papers has been retracted so far. There is no significant change to the reference list. | Pages 32 - 36 |
| **Reviewer #4** | | | |
| 2 | There are some minor typographical and grammatical mistakes that need correction for better clarity (such as sentence construction in the abstract, pluralization issues, and formal wording in response letters). These issues are relatively minor and do not hinder comprehension. | All typos and grammatical errors have been taken care of. We have improved on the grammar and the quality of the entire manuscript | All through the manuscript |
| 3 | Kindly examine the manuscript for minor typographical and grammatical errors, particularly in the abstract and the response to reviewers, to improve clarity further. | All typos and grammatical errors have been eliminated. We have improved on the grammar and the quality of the manuscript. The clarity of the manuscript | All through the manuscript |

| | | | |
|---|---|---|---|
| | | has been improved. | |
| 4 | Confirm that all supporting information files are adequately captioned and cited in the main text, following PLOS ONE guidelines. | In line with PLOS ONE guidelines, all supporting information files are adequately captioned and cited in the main text. | Page 36 |
| 6 | Please ensure that your manuscript meets PLOS ONE's style requirements, including those for file naming. The PLOS ONE style templates can be found at https://journals.plos.org/plosone/s/file?id=wjVg/ PLOSOne_formatting_sample_main_body.pdf and https://journals.plos.org/plosone/s/file?id=ba62/ PLOSOne_formatting_sample_title_authors_affiliations.pdf | The manuscript has been formatted to meet PLOS ONE's style requirements, including those for file naming. | All through the manuscript |
