# Supplementary material for: Geospatial assessment of household water, sanitation and hygiene conditions and associated factors in Nigeria: A causal relationship model
Source: PLoS One. 2025 Aug 14;20(8):e0330167. doi: 10.1371/journal.pone.0330167 (PMC12352836; doi:10.1371/journal.pone.0330167)
Supplement: Attachment — Submitted filename: Authors_Response.docx [file pone.0330167.s003.docx]

**GEOSPATIAL ASSESSMENT OF HOUSEHOLD WATER, SANITATION AND HYGIENE CONDITIONS AND ASSOCIATED FACTORS IN NIGERIA: A CAUSAL RELATIONSHIP MODEL**

Summarized authors’ response: We have made all the corrections in accordance with the Editor’s and Reviewers’ suggestions. The paper has been formatted in line with the guidelines of the Journal. The modified/added sentences are colored red.

| **S/No.** | **Academic Editor’s Comments** | **Changes Made** | **Page Number** |
| --- | --- | --- | --- |
| 1 | Please note that your Data Availability Statement is currently missing…..  We therefore suggest that you provide this information now, though we will not hold up the peer review process if you are unable | Data availability statement has been added to the manuscript |  |
| 2 | Regarding Figs 1, 4, 6, 7, 8, 9, 10, and 11 - please state what program(s) were used to create these maps | The program used for map creation has been stated |  |
| 3 | Additionally, we would like to request that your figure files are modified at this time to meet PLOS One's submission criteria. We request that all panels of a figure are submitted together as one file per figure | All panels of figure are now presented as one file per figure |  |
| 4 | Please ensure that your manuscript meets PLOS ONE's  style requirements, including those for file naming. The PLOS ONE style templates can be found at <https://journals.plos.org/plosone/s/file?id=wjVg/>  PLOSOne_formatting_sample_main_body.pdf and <https://journals.plos.org/plosone/s/file?id=ba62/>  PLOSOne_formatting_sample_title_authors_affiliations.pdf | The manuscript has been formatted to meet PLOS ONE’s style requirements, including those for file naming. | All through the manuscript |
| 5 | Please include your full ethics statement in the ‘Methods’ section of your manuscript file. In your statement, please include the full name of the IRB or ethics committee who approved or waived your study, as well as whether or not you obtained informed written or verbal consent. If consent was waived for your study, please include this information in your statement as well. | Ethics statement has been included in the method section | Page 5 (Lines 131 - 138) |
| 6 | We note that Figure 1, 4, 5, 6, 7, 8, 9, 10, and 11 in your submission contain [map/satellite] images which may be copyrighted. All PLOS content is published under the Creative Commons Attribution License (CC BY 4.0), which means that the manuscript, images, and Supporting Information files will be freely available online, and any third party is permitted to access, download, copy, distribute, and use these materials in any way, even commercially, with proper attribution. For these reasons, we cannot publish previously copyrighted maps or satellite images created using proprietary data, such as Google software (Google Maps, Street View, and Earth). For more information, see our copyright guidelines: http://journals.plos.org/plosone/s/licenses-and-copyright.  We require you to either (1) present written permission from the copyright holder to publish these figures specifically under the CC BY 4.0 license, or (2) remove the figures from your submission:  a. You may seek permission from the original copyright holder of Figure 1, 4, 5, 6, 7, 8, 9, 10, and 11 to publish the content specifically under the CC BY 4.0 license.  We recommend that you contact the original copyright holder with the Content Permission Form (http://journals.plos.org/plosone/s/file?id=7c09/content-permission-form.pdf) and the following text:  “I request permission for the open-access journal PLOS ONE to publish XXX under the Creative Commons Attribution License (CCAL) CC BY 4.0 (http://creativecommons.org/licenses/by/4.0/). Please be aware that this license allows unrestricted use and distribution, even commercially, by third parties. Please reply and provide explicit written permission to publish XXX under a CC BY license and complete the attached form.”  Please upload the completed Content Permission Form or other proof of granted permissions as an "Other" file with your submission.  In the figure caption of the copyrighted figure, please include the following text: “Reprinted from [ref] under a CC BY license, with permission from [name of publisher], original copyright [original copyright year].”  b. If you are unable to obtain permission from the original copyright holder to publish these figures under the CC BY 4.0 license or if the copyright holder’s requirements are incompatible with the CC BY 4.0 license, please either i) remove the figure or ii) supply a replacement figure that complies with the CC BY 4.0 license. Please check copyright information on all replacement figures and update the figure caption with source information. If applicable, please specify in the figure caption text when a figure is similar but not identical to the original image and is therefore for illustrative purposes only.  The following resources for replacing copyrighted map figures may be helpful:  USGS National Map Viewer (public domain): http://viewer.nationalmap.gov/viewer/  The Gateway to Astronaut Photography of Earth (public domain): http://eol.jsc.nasa.gov/sseop/clickmap/  Maps at the CIA (public domain): https://www.cia.gov/library/publications/the-world-factbook/index.html and https://www.cia.gov/library/publications/cia-maps-publications/index.html  NASA Earth Observatory (public domain): http://earthobservatory.nasa.gov/  Landsat: http://landsat.visibleearth.nasa.gov/  USGS EROS (Earth Resources Observatory and Science (EROS) Center) (public domain): http://eros.usgs.gov/#  Natural Earth (public domain): http://www.naturalearthdata.com/ | All figures were originally generated by the authors and none of them have been copyrighted. We do not need to take permission from anyone. All the figures and tables presented in the manuscript are genuine, original and none of them have been published, used or appeared in any form. The figures are exclusively generated for this study. The figures are geospatial expressions of the analyses results. | All figures are original, and were all produced by the authors strictly for this manuscript |
| 7 | Please include captions for your Supporting Information files at the end of your manuscript, and update any in-text citations to match accordingly. Please see our Supporting Information guidelines for more information: http://journals.plos.org/plosone/s/supporting-information. | The Supporting Information files have been listed | Page 36 |
| 8 | Please review your reference list to ensure that it is complete and correct. If you have cited papers that have been retracted, please include the rationale for doing so in the manuscript text, or remove these references and replace them with relevant current references. Any changes to the reference list should be mentioned in the rebuttal letter that accompanies your revised manuscript. If you need to cite a retracted article, indicate the article’s retracted status in the References list and also include a citation and full reference for the retraction notice. | We have ensured that only cited references are listed. None of the cited papers has been retracted so far. There is no significant change to the reference list. We only introduced two additional references |  |
| Reviewers’ Comments | | | |
| **Reviewer #1**  This manuscript is very rich in content, with complete data and correct statistical methods. In Nigeria, Water, Sanitation, and Hygiene (WASH) conditions are determined by financial status, level of education, living conditions, and the gender of the household head. Since my research field differs from the content described in this manuscript, some issues need to be adjusted or clarified during the review process. | | | |
| 1 | The manuscript determines the size of a household based on the number of household members, with households of more than 6 people classified as large-scale and those with 6 or fewer people as small-scale. Where does the number 6 come from? Is there no category for medium-sized households under this classification? | Thank you for your comment. Nigerian population policy (2007) recommended a maximum of 4 children per couple resulting in 6 household members. Since household size is synonymous to family size in Nigeria, our study categorized a household size of 6 or less as within the recommended household size while household size above 6 is considered to be above the recommended size. We have cited the population policy as our justification for the categorization. Also, since household sizes that are above 6 members were considered large, we did not see the reason for creating a category for medium-sized household. |  |
| 2 | 2.“The proportions were grouped into three equal categories, each category comprising 33.3% of the household population.” What is the specific classification method? Is it to calculate the total score for each household, divide it by the number of household members, rank the households by this number, and then classify the top third as high, the middle third as medium, and the bottom third as low in terms of household education level? | Thank you for pointing this out. We have clearly explained how the household education level was calculated in the manuscript. “We obtained the mean household education score by dividing the total household education score by the number of household members. The mean scores were divided into tertile such that the top third was categorized as high, the middle third as medium, and the bottom third as low.” | Pages 6 and 7 |
| 3 | The data collected in the manuscript is from 2018, and it is now 2025. Is the time span too large? | We understand the reviewer’s concern about the time of the data. Although the time of data collection is very important in research, especially when considering the current situation, it sometimes does not matter where appropriate data are not available and we do not know what ought to have been known. Our current study has presented what we ought to have known before now, but has been elusive to researchers. We believe our finding will serve as a baseline information or a reference point for more recent data. As of the time of this study, the DHS 2018 remained the most comprehensive, nationally representative and most recent data collected for Nigeria. It probably may be the last of the DHS datasets now that American government has stopped the DHS program globally. |  |
| 1 | **Reviewer #2:**  The manuscript is technically sound and the data support the conclusion. The statistical analysis has been appropriately and rigorously done. The manuscript is written in standard English, and in an intelligible manner. However, the author should check Figure 1. **On Figure one, there are two South-West geo-political zones. This should be corrected.** | We have replaced the map in Figure 1 with the right map that correctly identifies the six geo-political zones in Nigeria. | Figure 1 |
|  | **Reviewer #3:**  The paper is very interesting by providing spatial method in analysing household factors on WASH access. The paper has been written very thorough and complete, but there are some items can be improved as follow: | | |
| 1 | The paper is very long, perhaps can be considered to reduce the word count. | We have managed to reduce the word count significantly |  |
| 2 | Figure 2 and Table 5. Would it be possible to explain what does the values in the table representing for? Is it the numeric score of WASH access and household factors? Is each variable also analysed as binary variable (improved vs unimproved) | Figure 2 and Table 5 present the prevalence of the examined individual household factors among the sampled households in Nigeria. They showcase the percentage of the sampled households that fall under the examined household variables. Explanation has been given to this effect in the manuscript. The background to these statistical values is presented in the first paragraph of ‘Background characteristics of the households’ under the Results section in Pages 12 and 15 | Pages 21 (Lines 12-24) and 22 (Lines 1-5) |
| 3 | Figure 3. Is it possible to have the cartogram plotting on the geographical boundaries? It hard to understand which circle belong to which area. | Cartogram plotting can only be done for a geographical boundary (i.e, a country) that is sub-divided into smaller spatial entities (i.e., states). The algorithm considers the centroid coordinates of the states’ geographical areas. |  |
| 4 | Page 19. “However, results show that HBMWQ and HWRR exhibit stronger causal relationships with UN compared to HWLLE and HWRR.” **Is UN mean UW**? | The “UN” is rather “UH” which means unimproved hygiene | Page 18 (Line 404) |
| 5 | Table 8. UW has been explained in the text. But US and UH seems missing from the explanation | Thank you!  This has been addressed | Page 19 (Line 431) |
| 6 | Page 21: “This implies that the majority of married couples usually stay married till old age”. IS this relevant to the WASH access? | Thank you for the comment. We have expunged the sentence |  |
| 7 | Page 21: “We observe that the distribution of older adults has a similar spatial pattern to that of crime rate and social restiveness in Nigeria. For instance, the North East has been the epicentre of insurgency and banditry for more than a decade and the most vulnerable people are the elderly persons who are physically weak as a result of old age. Whereas in the South-South, the ongoing environmental degradation as a result of oil pollution and the consequent deterioration in public health could result in low life expectancy in oil-producing areas.” This part perhaps needs to be discussed closer in later part in 1st paragraph in page 24. | We have provided additional discussion | Page 22 (Lines 489 – 494) |
| 8 | Page 21: “We observed that unimproved hygiene is a general but ugly phenomenon that is rampant among people across all socioeconomic strata in Nigeria.” Is there any impact from COVID-19 pandemic massive promotion of hand hygiene to the improvement of hygiene practice in Nigeria? | Since the data were collected prior the COVID-19, it is difficult to comment on the impact of COVID-19 on hand hygiene in Nigeria. |  |
| 9 | Page 22: “Such suspected influencing factors could be more environmental conditions rather than household factors.” Beside environmental factors, local state policy and programs, experience with WASH program in particular state may also influence WASH access and behavior. | We agree with your comment on the role of local or state policy on WASH access/behavior. The comment has been added to our discussion. Thank you. |  |
| 10 | Page 26: “In this study, we present an elite analytical method that not only establishes a causal relationship between household factors and WASH conditions but also models the spatial variability of the causal relationship as well as the spatial pattern of individual household parameter influence on WASH conditions.” Would you please elaborate more, what tis the new perspective or understanding can be contributed using these spatial analytical methods compare to standards statistical analysis? | The contributions of our methods have been elaborated in page 30 | Page 30 (Lines 673 – 687) |
| 11 | In discussion section there are some redundancies in explaining influence each household factors (FHH, economic, education etc factors). It could be reorganised to have more concise explanation. | We have resolved this. We have eliminated all redundancies |  |
| 12 | Conclusion has been made based on the findings, but perhaps conclusion on the benefit of using the spatial analysis is worth to be included. | The benefit of spatial analyses has been included in the conclusion. | Page 31 (Pages 713-714) |
| 13 | There are many studies have similar findings in regards to the household’s factors influence of WASH access which can be refer in discussion section, which is currently lacking | Our focus is on the modeling of the influence of household factors on WASH conditions. The study is particular about the spatial variability of the causal relationships between household factors and WASH conditions in Nigeria. The study pays attention to the spatial variability of individual household parameter influence on WASH conditions in Nigeria.  Our study is the first of its kind. Thus, our approach, methods, focus, results, and findings are completely different from that of the existing studies. |  |
|  | **Reviewer #4:** |  |  |
| 1 | Clarity on "Unimproved Hygiene": The manuscript frequently mentions "unimproved hygiene." Please define this term clearly in the introduction or methods section. Specifying what constitutes "unimproved hygiene" will help readers better understand the scope of the study. | Thank you for your comment on this. We have included the definition of water, sanitation and hygiene in the introduction. The unimproved WASH conditions have been explained in the method section. | Page 6 (Lines 140 – 158) |
| 2 | Justification for Statistical Methods: While the methods are mentioned, provide a brief justification for using spatial statistics in addition to non-spatial methods. Why is a spatial approach particularly valuable for this research question? Expand on why spatial regression was appropriate. | Spatial statistics is particularly valuable for this research because it offers us the opportunity to model the spatial variability of the causal relationship between WASH conditions and household factors over the study area. In addition, the adoption of local spatial regression model enables us to model and visualize location-specific parameter influence as well as the variability of the model performance. | Page 7 (Lines 176 – 178)  Page 30 (Lines 678 – 687) |
| 4 | Interpretation of DoD: Expand on the interpretation of the Degree of Dependence (DoD_j) values. While the results state that residency type has a higher DoD_j for water source choice, explain the practical implications of this finding. What does a DoD_j of 0.998 mean in terms of policy or interventions? | DoD_j measure the dependence of a response variable on a predictor variable. In this case, the most influencing variable in a spatial regression model may not be the strongest predictor variable. In the case of UW prediction for example, highest Adjusted R2 value was recorded for HBMWQ while the highest DoD_j is recorded for HWRR. The interpretation is that the consumption of unimproved water is common among the poor, but rampant in the rural areas due to low accessibility to improved water. DoD_j of 0.998 means that WASH conditions have very high dependency on standard of living of the rural populace in Nigeria. | Page 25 (Lines 554 – 574) |
| 5 | Limitations: The authors acknowledge that the best predictive models only account for 76% of the variance. Elaborate on the potential factors that might explain the remaining variance and discuss the implications of these unmeasured factors. What specific data limitations might affect the conclusions? | We have elaborated on the unmeasured influencing factors. And specific data limitation and the attendant limitations have been added to the manuscript I Page 32 | Page 29 (Lines 663 – 668) |
| 6 | English Language and Grammar: While generally well-written, the manuscript could benefit from a careful review by a native English speaker to address minor grammatical issues and improve clarity. For example, in the abstract, "...morbidity of school-age children" could be rephrased for smoother reading. Also, check for consistency in terminology. | Thank you for your observation. We have revised the manuscript to address all grammatical issues. | The entire manuscript |
| 7 | Policy Recommendations: While the policy recommendations are relevant, make them more specific and actionable. For example, instead of just stating "the need for more focused policy action," suggest specific types of interventions, target populations, or potential partnerships. | We have suggested specific types of interventions, target population and potential partnerships. Page 34 | Page 32 (720 -725) |
| 8 | Literature Review: Consider expanding the literature review to include more recent studies on WASH interventions in Nigeria and similar contexts. This will help contextualize the study's findings and demonstrate its contribution to the existing body of knowledge. | We have added two recent references. However, the focus of this study is not on WASH intervention. Rather, our study focuses on the influence of household factors on WASH conditions. The contributions of this study have been well elucidated in the discussion section | Page 33 (Lines 774 – 776),  Page 34 (804 – 806) |
|  | **Reviewer #5:** |  |  |
| 1 | The abstract of this paper can be shortened. The literature gap is not evident in the introduction section. I advise author to elaborate his contribution in literature. | The abstract has been revised and shortened.  The research and knowledge gaps that are addressed by this study have been well presented in the introduction section. The emphasis of the study is on modeling the influence of household factors on WASH conditions, adopting spatial statistics and geospatial technology. The novelty of our method has been emphasized in the discussion section | Page 1 (Lines 14 – 29)  Page 4 (92 - 111) |
| 2 | I suggest adding a table, describing the variables used in this research along with their descriptive statistics (in variable measurement section or appendices). | The variables used in this study have been described in the method section. We have also presented the descriptive statistics in the supplementary tables 1 and 2. Since the study is household-based, not individual based, we have presented the household characteristics in each state in the supplementary table 1. | S1 file |
| 3 | Author should elaborate the choice of using techniques like Global regression models, OLS, Local Regression model and Multiscale geographically weighted regression, their importance in the context of this research. | This has been taken care of in Pages 7 and 30 | Page 7 (Lines 175 – 178)  Page 30 (Lines 679 – 687) |
| 4 | Inconsistence in table notes, a few tables in the manuscript lack table note for example 7 and 8. | Thank you for flagging this. We have added the footnotes | Page 20 |
| 5 | The discussion section is well written, but conclusion of this research is too short, I recommend author to rewrite conclusion of this manuscript by taking research practice in consideration. | Thank you. We have updated the conclusion of the findings | Page 31 (Lines 713 - 714) |
|  | **Reviewer #6** |  |  |
| 1 | Line numbers should be added for providing comments line by line from reviewers. | Thank you. We have added the line numbers |  |
| 2 | Sample design: Instead of referring sampling procedure in the DHS report, it is better to describe the type of sampling at the cluster and household levels. | We agree with the reviewer on the need for detailed sample design. However, since the data used for this study was a secondary data and the detailed description of the sample design has been published, we believe the publication can be cited to avoid overly lengthy manuscript. |  |
| 3 | How does a protected spring differ from an unprotected spring? | The word “spring” in “protected well and string” as well as “unprotected well and spring” may not be isolated. The protected well and string referred to well and spring water sources that were covered to prevent dirths and animals from contaminating the water, while the unprotected ones were uncovered. These were responses which were unique to DHS questions to accommodate all country-specific practices. |  |
| 4 | For education, does low education include primary and no education? | Education in this study was computed for all household members. Thus, it is the household level of education. We have explained how we computed the variable in the method section | Page 6 |
| 5 | How to define household size by 6? Is there any reason for using 6 as the cutoff point? | The use of 6 as the household size was based on Nigeria’s population policy which allows four children per couple. This has been justified in the method section. | Page 6 |
| 6 | In Table 4, adjusted R2 and AIC should be described separately for model fitness because lower AIC values indicate better fitness. In this analysis, MGWR yields better model statistics because of higher adjusted R2 and lower AIC. | This suggestion has been taken care of already in Page 15.  For clarity’s sake, Adjusted R2 and AIC values are better described together | Page 15 (Lines 318 – 336) |
| 7 | In the Local Regression Model, the Authors added Structural Equation Modelling (SEM), and Spatial Lag Models (SLM). What findings represent SEM in your findings? Do you mean it as a Spatial Error Model? | Thank you for spotting this. We have taken care of the error. SEM represent Spatial Error Model, while SLM represent Spatial Lag Model | Page 8 (Lines 197 - 198) |
| 8 | Another one is that it is unclear whether Spatial or Special Lag Models exist. Please answer thoroughly for clarification. | We are referring to ‘Spatial Lag Model’. It was an error and we have rectified it. | Page 8 (Lines 206 and 207) |
| 9 | In supporting information 1, binary logistic regression was shown where independent variables were mentioned as household characteristics. Under the analytical procedure (page 7), household factors are HMWQ, HLLE, HRR, and FHH. Therefore, these definitions are not clearly cut. | The same household characteristics listed in the supplementary table were the household factors listed with their acronyms in the method section. Since our study utilized spatial analysis, the independent variables were not used as individual characteristics of the respondents but as aggregates (proportions) for each state. |  |
| 10 | Another question is why regression analysis at the individual level is added in supporting information. Your study objective is to examine the relationship between household factors and WASH in terms of spatial context. | The results presented in the supplementary tables were at state level which were used in conjunction with the GIS raster data for the spatial analysis. |  |
| 11 | For Geospatial analysis, the data set should be representative of the entire population like Census data. In the DHS data, the sampling is a probability proportionate method and so although it is a nationwide survey, the target population is not covered for geospatial analysis. Therefore, authors are suggested to add this limitation as a discussion point. | We understand the reviewer’s concern. The DHS data was a household-based nationally representative data. The survey collected needed and relevant information about women (DHS calls it individual recode), men (DHS calls it men’s recode), under-five children (kids recode), birth history (birth recode), couple (couple’s recode), the entire household member (people’s recode), and the household itself (household recode). This study is based on the household data (household recode). So, the household is the focus of this study. Although the DHS is not a census data, since aggregate data for the geographical units could be obtained and the raster data are available, geospatial analysis could be conducted. |  |
| 12 | The conclusion should be based on the research findings. It is suggested to modify the statement “Households with these characteristics may not be mentally, physically, and economically prepared to provide improved WASH for the members due to a possible lack of information about the health consequences, financial incapacity and poverty of the environment in which they live” because this study does not include analysis on mental status and access to information about the health consequences. | Thank you. The reviewer’s concern has been addressed. | Pages 31 - 32 |
|  |  |  |  |
